# Supplementary material for: Identification of a distal RXFP1 gene enhancer with differential activity in fibrotic lung fibroblasts involving AP-1
Source: PLoS One. 2021 Dec 31;16(12):e0254466. doi: 10.1371/journal.pone.0254466 (PMC8719731; doi:10.1371/journal.pone.0254466)
Supplement: S1 Table — (DOCX) [file pone.0254466.s001.docx]

**Table S1. Genomic location and size of cloned *RXFP1* fragments**

| Plasmid | Genomic Location (GRCh38/hg38) | Relative Location (TSS) | Size (bp) |
| --- | --- | --- | --- |
|  | **Proximal*** | | |
| Pcore | chr4:158521569-158521812 | -145 to 98 | 244 |
| PE | chr4:158520512-158521875 | -1202 to 161 | 1364 |
| PE-D1 | chr4:158520786-158521875 | -928 to 161 | 1090 |
| PE-D2 | chr4:158521212-158521875 | -502 to 161 | 664 |
|  | **Distal^#^** | | |
| Dcore | chr4:158315169-158315401 | -142 to 90 | 233 |
| DE | chr4:158313153-158316282 | -2158 to 971 | 3130 |
| DE-D1 | chr4:158313723-158316282 | -1588 to 971 | 2560 |
| DE-D2 | chr4:158314360-158316282 | -951 to 971 | 1923 |
| DE-D3 | chr4:158315050-158316282 | -261 to 971 | 1233 |
| DE-D4 | chr4:158315711-158316282 | 400 to 971 | 572 |
| DE-D2toD3 | chr4:158314360-158315050 | -951 to -261 | 691 |
| DE-TFBS | chr4:158314636-158315243 | -675 to -68 | 608 |
| TFBS-D1 | chr4:158314727-158315243 | -584 to -68 | 517 |
| TFBS-D2 | chr4:158314819-158315243 | -492 to -68 | 425 |
| TFBS-D3 | chr4:158314923-158315243 | -388 to -68 | 321 |
| TFBS-D4 | chr4:158315050-158315243 | -261 to -68 | 194 |
| TFBS-D5 | chr4:158314636-158314750 | -675 to -561 | 115 |
| TFBS-D6 | chr4:158314636-158314839 | -675 to -472 | 204 |
| TFBS-D7 | chr4:158314636-158314942 | -675 to -369 | 307 |
| TFBS-D8 | chr4:158314636-158315069 | -675 to -242 | 434 |
| TFBS-C | chr4:158314727-158315069 | -584 to -242 | 343 |
| TFBS-CD1 | chr4:158314727-158314942 | -584 to -369 | 216 |
| TFBS-CD2 | chr4:158314783-158314942 | -528 to -369 | 160 |
| DE_M1 | chr4:158314727-158315069 | -584 to -242 | 343 |
| DE_M2 | chr4:158314727-158315069 | -584 to -242 | 343 |

* Short transcript: transcription start site (TSS): chr4:158,521,714

^#^ Long transcript: transcription start site (TSS): chr4:158,315,311
