# Supplementary material for: Identification of a distal RXFP1 gene enhancer with differential activity in fibrotic lung fibroblasts involving AP-1
Source: PLoS One. 2021 Dec 31;16(12):e0254466. doi: 10.1371/journal.pone.0254466 (PMC8719731; doi:10.1371/journal.pone.0254466)
Supplement: S2 Table — (DOCX) [file pone.0254466.s002.docx]

Table S2. Demographic and clinical characteristics of subjects from the LGRC study

|  | **FOS*** | | | **JUN#** | | |
| --- | --- | --- | --- | --- | --- | --- |
|  | **Ctrl** | **IPF** | ***P* value  (Ctrl vs. IPF)** | **Ctrl** | **IPF** | ***P* value  (Ctrl vs. IPF)** |
| Number of subjects, n (%) | 108 | 160 |  | 22 | 22 |  |
| Gender |  |  |  |  |  |  |
| Male | 49 (45) | 109 (68) | <0.000 | 13 (59) | 17 (77) | 0.042 |
| Female | 59 (55) | 50 (32) |  | 9 (41) | 5 (23) |  |
| Age, yr, mean (SD) | 63.62 (11.35) | 64.10 (8.51) | 0.693 | 62.18 (9.61) | 63.45 (8.67) | 0.647 |
| Smoking history, n (%) |  |  |  |  |  |  |
| Current | 2 (2) | 2 (1) | <0.000 | 1 (4) | -- | 0.925 |
| Ever | 63 (58) | 96 (60) |  | 16 (73) | 19 (86) |  |
| Never | 32 (30) | 58 (36) |  | 3 (14) | 3 (14) |  |
| N/A | 11 (10) | 4 (3) |  | 2 (9) | -- |  |
| Pulmonary function, mean(SD) |  |  |  |  |  |  |
| FEV_1_ predict, % predicted | 95.02 (12.62) | 71.02 (18.02) | <0.000 | 92.14 (11.54) | 65.91 (16.19) | <0.000 |
| FVC predict, % predicted | 94.36 (13.13) | 64.30 (16.72) | <0.000 | 90.04 (14.75) | 59.82 (13.27) | <0.000 |
| D_LCO_, % predicted | 84.05 (16.69) | 48.03 (18.44) | <0.000 | 81.65 (17.66) | 36.90 (16.78) | <0.000 |

*gene expression was analyzed using microarray; #gene expression was analyzed using bulk RNA sequencing.
