# Supplementary material for: Identification of a distal RXFP1 gene enhancer with differential activity in fibrotic lung fibroblasts involving AP-1
Source: PLoS One. 2021 Dec 31;16(12):e0254466. doi: 10.1371/journal.pone.0254466 (PMC8719731; doi:10.1371/journal.pone.0254466)
Supplement: S1 Fig — (A) chromatin characteristics associated with active transcriptional regulation including H3K4Me1, H3K27Ac, H3K4Me3 and DNAse sensitivity clusters using the Encyclopedia of DNA Elements (ENCODE) histone ChIP data tracts in the UCSC genome browser (https://genome.ucsc.edu/). (B) Transcription factors identified using the UCSC Genome Browser for the 608bp enhancer region. The narrowed 343bp enhancer was boxed in red. Nucleotide locations of the two AP-1 sites are labeled. The colored boxes with specific numbers correspond to specific transcription factors and detailed in (C). (PDF) [file pone.0254466.s003.pdf]

(A)

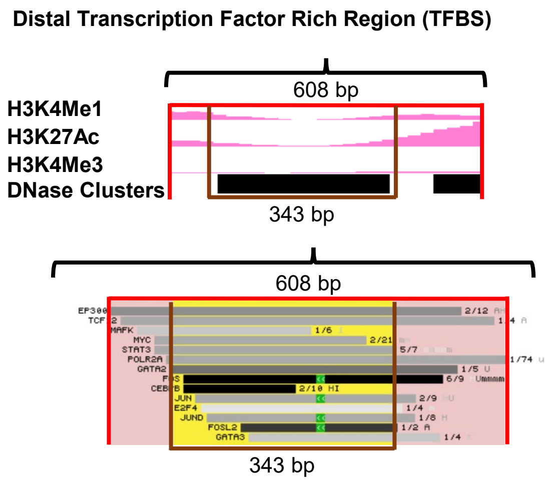


(B)

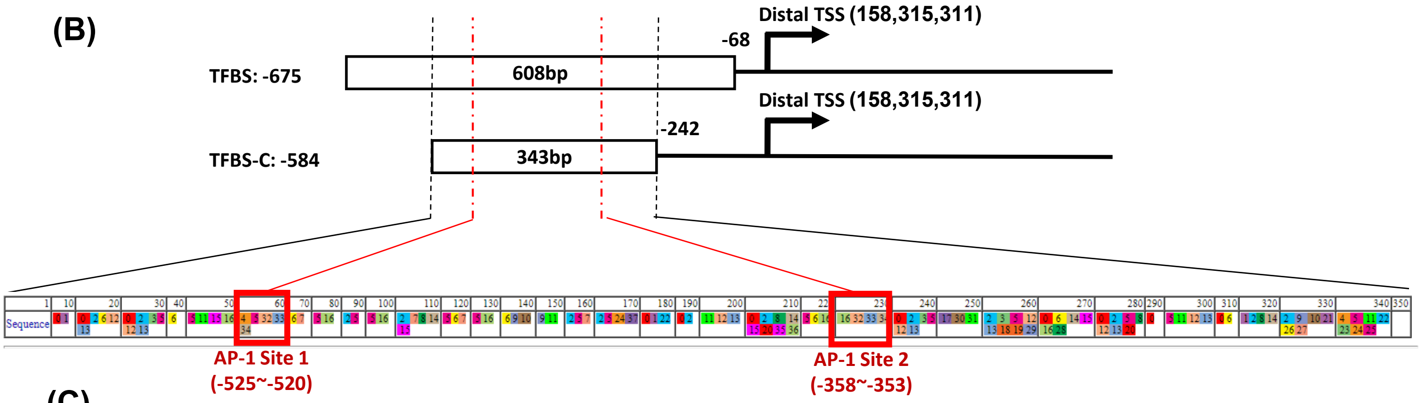


(C)

| | | | | | | | | | | | | | | | |
|---|---|---|---|---|---|---|---|---|---|---|---|---|---|---|---|
| 0 | FOXP3 | 1 | C-Myb | 2 | C/EBPbeta | 3 | C/EBPalpha | 4 | YY1 | 5 | GR-beta | 6 | TFIID | 7 | HNF-3alpha |
| 8 | NFI/CTF | 9 | GR-alpha | 10 | AP-2alphaA | 11 | TFIL-I | 12 | PRB | 13 | PR-A | 14 | NF-1 | 15 | ENKTF-1 |
| 16 | XBP-1 | 17 | GATA-3 | 18 | HoXD9 | 19 | HOXD10 | 20 | GR | 21 | NF-AT2 | 22 | STAT4 | 23 | NF-AT1 |
| 24 | C-Ets-1 | 25 | NF-AT1 | 26 | Pax-5 | 27 | p53 | 28 | VDR | 29 | PXR-1-RXR-alpha | 30 | GATA2 | 31 | GATA-1 |
| 32 | AP-1 | 33 | C-JUN | 34 | C-FOS | 35 | RXR-alpha | 36 | RAR-beta | 37 | Elk-1 | | | | |

**S1 Fig.** Transcription factor binding sites within distal enhancer region of the RXFP1
