# Supplementary material for: Identification of a distal RXFP1 gene enhancer with differential activity in fibrotic lung fibroblasts involving AP-1
Source: PLoS One. 2021 Dec 31;16(12):e0254466. doi: 10.1371/journal.pone.0254466 (PMC8719731; doi:10.1371/journal.pone.0254466)
Supplement: S2 Fig — (A) Original photos for the EMSA analysis showing in Fig 5A (left top) and 5B (right). The (A) left bottom was not used in the main text but was for an experiment performed at the same time as that for Fig 5A. Therefore, we included it for data integrity. The arrow indicates an artifact as it is located in between the two lanes. (B) Independent EMSA analysis using nuclear proteins from control and IPF and not a part of the main figures. Nuclear proteins isolated from control (CL) and IPF lung fibroblasts were used for the binding assay with a biotin-labeled 37-base pair double-stranded oligonucleotide containing the AP-1-binding motif site 2 of the RXFP1 enhancer. The unlabeled wildtype (wt) or mutated (mut) AP-1 site probe at 50-fold of labeled wt RXFP1 probe were used as unlabeled competitor for the specific binding competition assays. Supershift analyses with antibodies specific for JUN, FOS, JUNB and FOSL1 or control IgG are shown. The AP-1 specific complex is labeled as “complex A”. The supershifted complex with antibody specific for JUN is labeled as “complex B”. Unbound free labeled probe band is marked as “free probe”. (PDF) [file pone.0254466.s004.pdf]

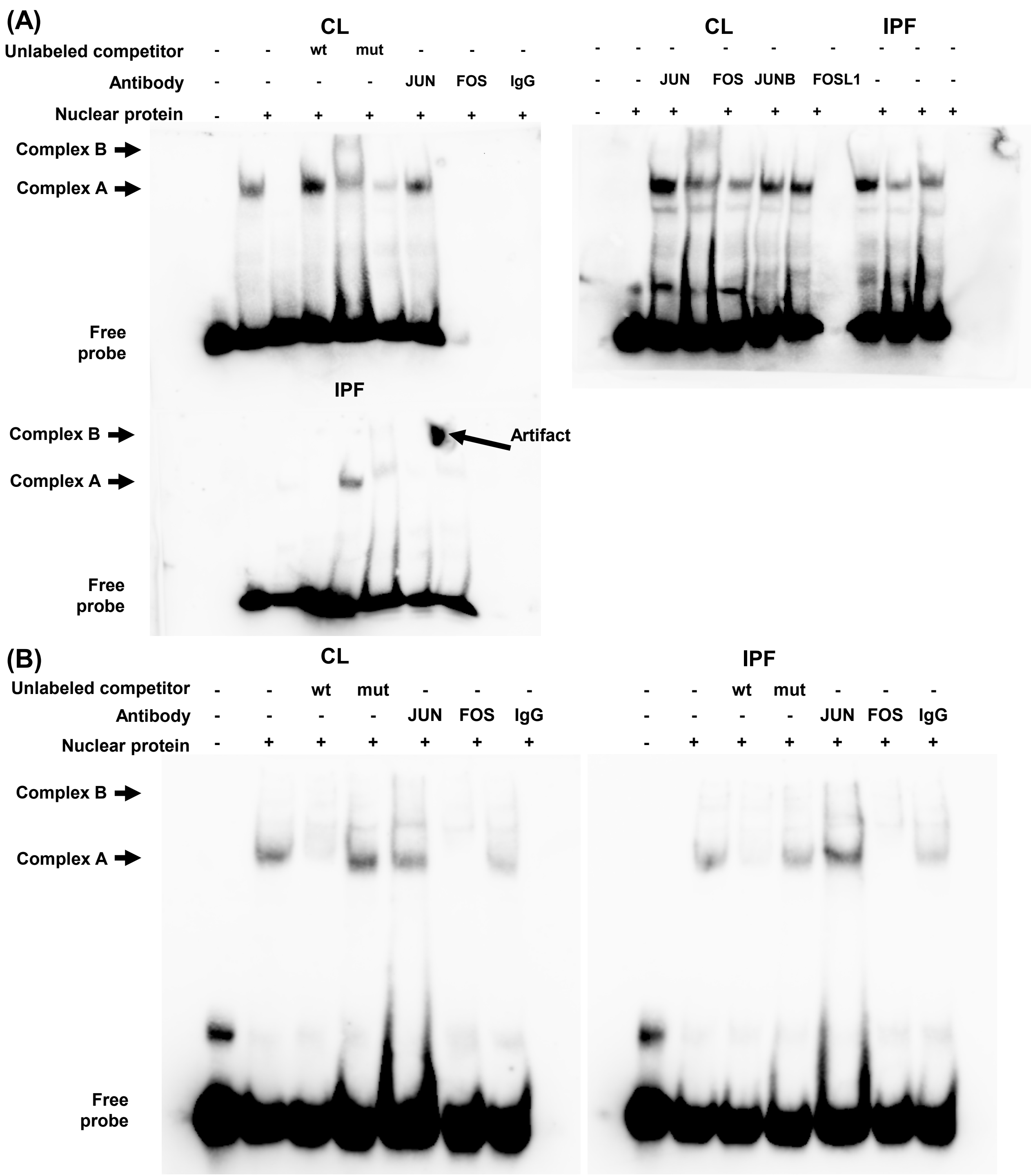

S2 Fig. Original pictures for EMSA in Fig 5A and 5B and additional EMSA using nuclear protein from control and IPF fibroblasts
