# Supplementary figures and images for: Identification of a distal RXFP1 gene enhancer with differential activity in fibrotic lung fibroblasts involving AP-1
Source: PLoS One. 2021 Dec 31;16(12):e0254466. doi: 10.1371/journal.pone.0254466 (PMC8719731; doi:10.1371/journal.pone.0254466)

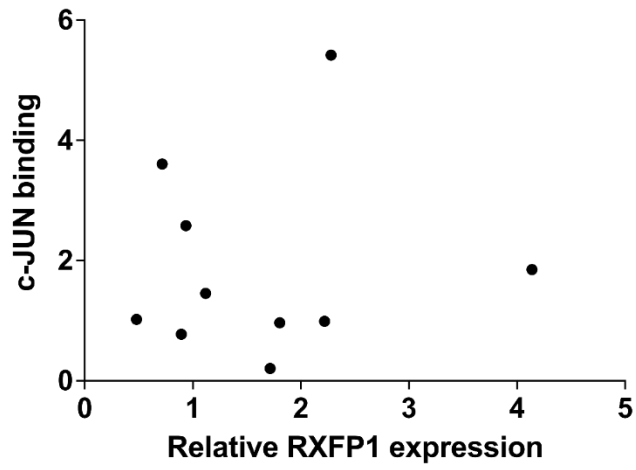

**S3 Fig.** Correlation of JUN binding in ChIP assay to RXFP1 gene expression by qPCR

Supplement: S3 Fig — RXFP1 gene expression was analyzed using a quantitative PCR (qPCR) with a Taqman probe (Hs01073141_m1) and standard protocol. The qPCR was analyzed using a QuantStudio 5 System (Applied Biosystem Inc.). Correlation of JUN binding analyzed by the ChIP analysis on Fig 5C and 5D was performed using the qPCR results in Graphpad 7 and no correlation was observed. (PDF) [file pone.0254466.s005.pdf]

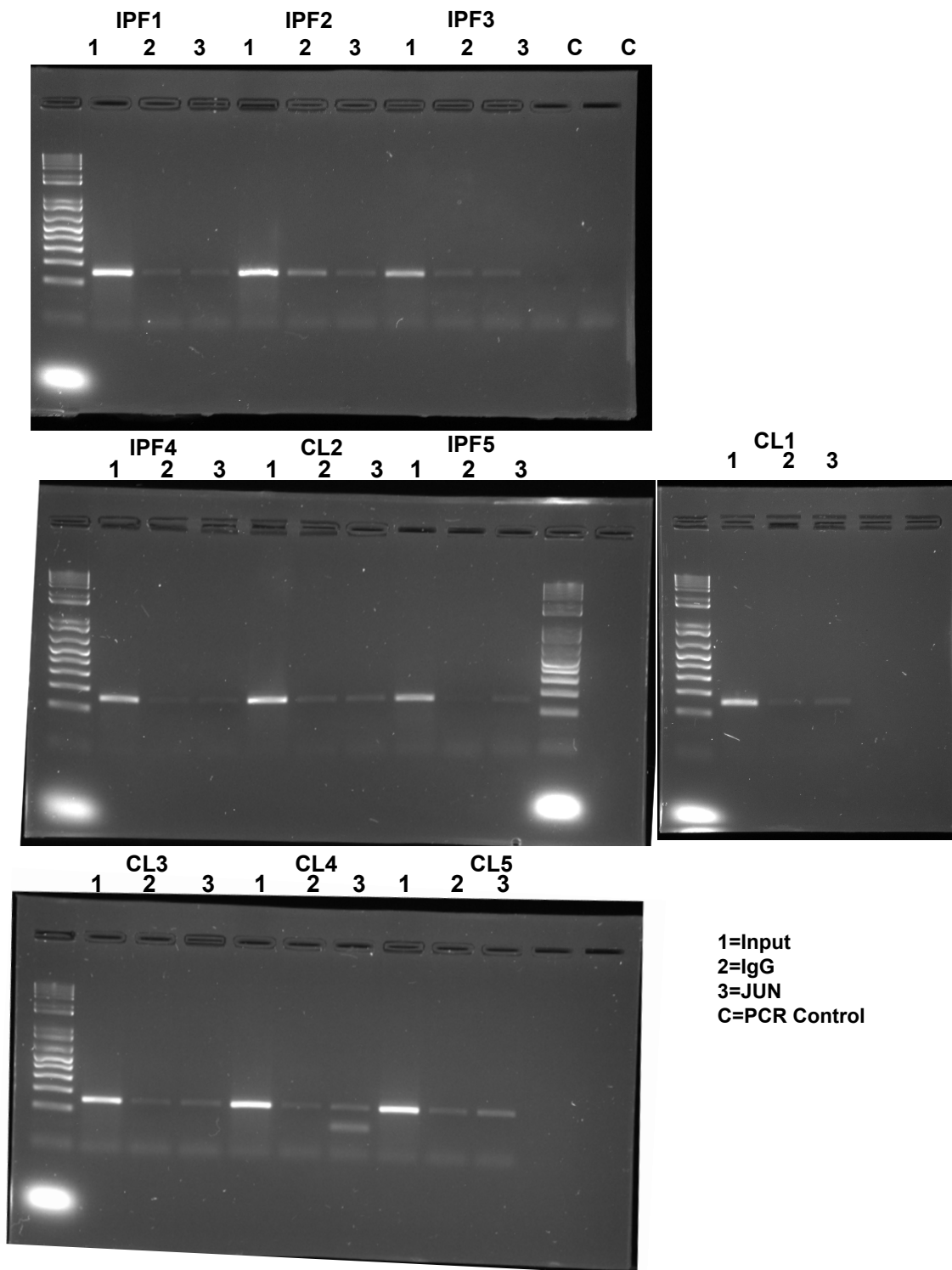

**S5 Fig.** Original DNA agarose gel images for the ChIP analysis Fig 5C

Supplement: S5 Fig — Agarose gel images from the gel electrophoresis for the Chromatin Immunoprecipitation (ChIP) analysis shown in Fig 5C. 1 = input, 2 = IgG, 3 = JUN antibody, C = PCR Control, CL = control fibroblast. Molecular weight markers are included for each gel. (PDF) [file pone.0254466.s007.pdf]

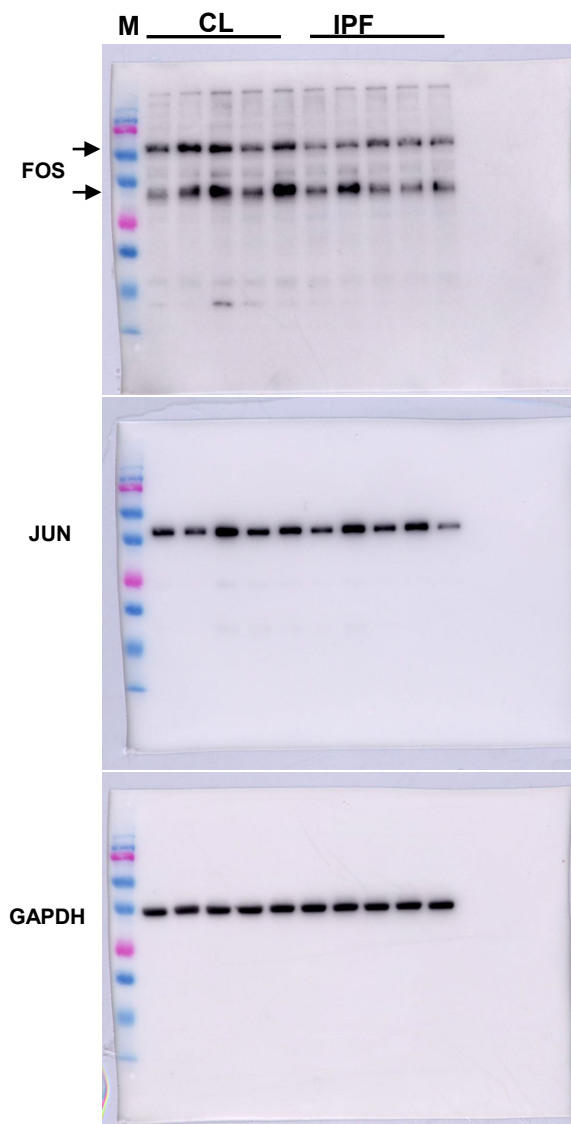

**S6 Fig.** Original Western blot images for Fig 6E

Supplement: S6 Fig — The bands specific for FOS, JUN and GAPDH are shown. (PDF) [file pone.0254466.s008.pdf]
