# Supplementary material for: Identification of a distal RXFP1 gene enhancer with differential activity in fibrotic lung fibroblasts involving AP-1
Source: PLoS One. 2021 Dec 31;16(12):e0254466. doi: 10.1371/journal.pone.0254466 (PMC8719731; doi:10.1371/journal.pone.0254466)
Supplement: S4 Fig — (A) Lung tissue expression levels of FOS in control and IPF (n = 22 for each) analyzed using bulk RNA sequencing from the publicly available Lung Genomics Research Consortium (LGRC) gene expression dataset (GEO accession GSE47460; http://www.lung-genomics.org/). Correlation of FOS (right) gene expression levels with RXFP1 was analyzed in IPF lungs (22 subjects) using linear regression and the R2 and p-value are shown. (B) Correlation of FOSL2 gene expression levels with RXFP1 was analyzed with microarray in IPF lungs (160 subjects) using linear regression and the R2 and p-value are shown. (PDF) [file pone.0254466.s006.pdf]

(A)

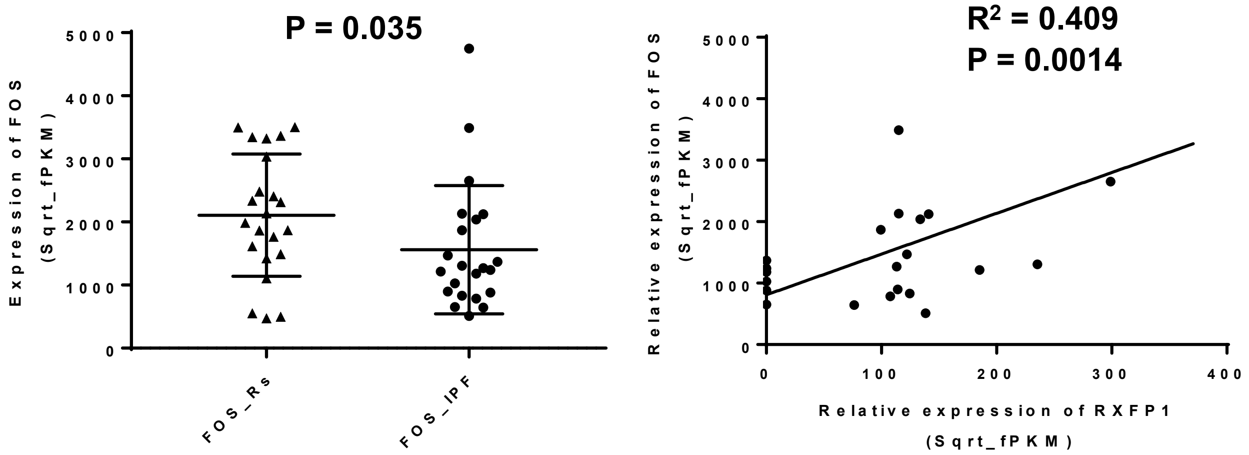

(B)

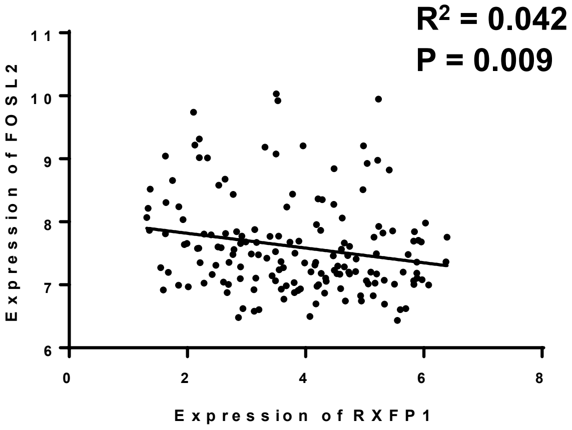

S4 Fig. FOS gene expression in IPF and control lungs and correlation of FOS and FOSL2 gene expression with RXFP1 in LGRC
